# Homoharringtonine is synergistically lethal with BCL-2 inhibitor APG-2575 in acute myeloid leukemia

**DOI:** 10.1186/s12967-022-03497-2

**Published:** 2022-07-06

**Authors:** Wenwen Wei, Shujuan Huang, Qing Ling, Shihui Mao, Yu Qian, Wenle Ye, Fenglin Li, Jiajia Pan, Xiangjie Lin, Jiansong Huang, Xin Huang, Yifan Zhai, Jie Sun, Jie Jin

**Affiliations:** 1 Department of Hematology, The First Affiliated Hospital, Zhejiang University School of Medicine, No. 79 Qingchun Road, Hangzhou, Zhejiang China; 2 Key Laboratory of Hematologic Malignancies, Diagnosis and Treatment, Hangzhou, Zhejiang China; 3 Zhejiang University Cancer Center, Hangzhou, Zhejiang China; 4 Zhejiang Provincial Clinical Research Center for Hematological Disorders, Hangzhou, Zhejiang China; 5 The First Affiliated Hospital of USTC, Division of Life Sciences and Medicine, University of Science and Technology of China, Hefei, Anhui China; 6 Ascentage Pharma (Suzhou) Co., Ltd, 68 Xingqing Street, Suzhou, China

**Keywords:** Acute myeloid leukemia, Homoharringtonine, APG-2575, MCL-1, GSK3β

## Abstract

**Background:**

Despite advances in targeted agent development, effective treatment of acute myeloid leukemia (AML) remains a major clinical challenge. The B-cell lymphoma-2 (BCL-2) inhibitor exhibited promising clinical activity in AML, acute lymphoblastic leukemia (ALL) and diffuse large B-cell lymphoma (DLBCL) treatment. APG-2575 is a novel BCL-2 selective inhibitor, which has demonstrated anti-tumor activity in hematologic malignancies. Homoharringtonine (HHT), an alkaloid, exhibited anti-AML activity.

**Methods:**

The synergistic effects of APG-2575 and HHT were studied in AML cell lines and primary samples. MTS was used to measure the cell viability. Annexin V/propidium iodide staining was used to measure the apoptosis rate by flow cytometry. AML cell xenografted mouse models were established to evaluate the anti-leukemic effect of BCL-2 inhibitor, HHT and their combination in vivo*.* Western blot was used to determine the expression of related proteins.

**Results:**

APG-2575 showed comparable anti-leukemic effect to the FDA-approved BCL-2 inhibitor ABT-199 in vitro and in vivo. Combined treatment of HHT with APG-2575 synergistically inhibited AML cell growth and engraftment. Mechanistically, HHT promoted degradation of myeloid cell leukemia-1 (MCL-1), which was reported to induce BCL-2 inhibitor resistant, through the PI3K/AKT/GSK3β signaling pathway.

**Conclusion:**

Our results provide an effective AML treatment strategy through combination of APG-2575 and HHT, which is worthy of further clinical research.

**Supplementary Information:**

The online version contains supplementary material available at 10.1186/s12967-022-03497-2.

## Background

Acute myeloid leukemia (AML) is a clonal aggressive hematological malignant disease with clinical and genetic heterogeneity [1, 2]. Although treatment of AML has made a significant achievement, the majority of patients experience a relapse of primary AML and develop resistance to chemotherapy [3]. In particular, elderly patients with AML are ineligible for intensive chemotherapy and their 5-year overall survival rate is only 5–15% [4]. It remains a major challenge in AML management due to refractoriness, relapse and drug resistance.

The B-cell lymphoma-2 (BCL‐2) is dysregulated in AML and its overexpression mediates therapeutic resistance and poor clinical prognosis, which makes BCL-2 an encouraging therapeutic target [5–7]. ABT-199 (venetoclax/GDC-0199), selectively targeting BCL-2, is a novel BCL-2 homology domains 3 (BH3) mimetic agent. In vitro studies had confirmed the remarkable anti-leukemic effect of ABT-199 in either chemotherapy-sensitive or chemotherapy-resistant AML cells, and more importantly, it was reported to eliminate leukemia stem cells [8, 9]. The combination of ABT-199 with hypomethylating agents (decitabine or azacitidine) achieved complete remission (CR) or CR with incomplete blood count recovery (CRi) in 67% of elderly patients with AML [10]. 54% of elderly AML adults who received combined regimen of ABT-199 and low-dose cytarabine (LDAC) achieved CR/CRi [11]. In addition, clinical trials of ABT-199 in combination with FMS-like tyrosine kinase 3 (FLT3) inhibitors, isocitrate dehydrogenase (IDH1/2) inhibitors and other agents on AML have been launched [12].

APG-2575 (lisaftoclax) is a BCL-2 selective inhibitor independently developed by Suzhou Ascentage Pharma, several clinical trials have been initiated in hematologic malignancies [13]. The results of the phase I trial (NCT03537482) showed that APG-2575 had anti-tumor activity and tolerable safety in patients with relapsed/refractory chronic lymphocytic leukemia (R/R CLL), small lymphocytic lymphoma (SLL) and other hematologic tumors [14]. APG-2575 combined with rituximab/ibrutinib or HHT/ azacytidine is currently being tested clinically for SLL/CLL or R/R AML (NCT04494503, NCT04501120). APG-2575 in combination with Bruton’s tyrosine kinase (BTK) inhibitor (ibrutinib) or MDM2-p53 inhibitor (APG-115) showed a synergistic anti-tumor effect in diffuse large B-cell lymphoma (DLBCL) [15]. In addition, combination therapy of APG-2575 and tyrosine kinase inhibitor (TKI) (olverembatinib/HQP1351) showed synergistic anti-leukemic effects in FLT3-ITD mutant AML [13]. Myeloid cell leukemia-1 (MCL-1) was highly expressed in multiple hematological malignancies [16], and was shown to mediate the development of hematological malignancies, including AML [17, 18]. Moreover, up-regulation of MCL-1 expression was observed during treatment with ABT-199, leading to the development of drug resistance [16, 19].

Homoharringtonine (HHT) is an alkaloid obtained from the *Cephalotaxus hainanensis*. It is successfully applied in the treatment of hematological diseases due to its effective anti-tumor activity [20, 21]. In our previous study, we found that HHT down-regulated the BCL-2 and MCL-1 expression [22], making us hypothesize combination of HHT with APG-2575 may enhance the response and reverse the APG-2575 resistance in AML cells.

In the present study, we evaluated the synergistic anti-AML effect of combined treatment of APG-2575 and HHT in AML. Our results revealed that the addition of HHT potentiated the anti-leukemic effect of APG-2575 in AML cell lines and primary AML cells in vitro and in vivo. Mechanistically, HHT inhibited the Phosphoinositide-3-Kinase/Protein kinase B/Glycogen synthase kinase-3 (PI3K/AKT/GSK3β) signaling pathway, leading to MCL-1 dual phosphorylated and degraded, which overcame the resistance of APG-2575 mediated by overexpressed MCL-1. In conclusion, our results support further exploring the effect of the combination regimen of HHT and APG-2575 in AML treatment.

## Materials and methods

### Materials

APG-2575 was provided by Ascentage Pharma (Suzhou) Co., Ltd and ABT-199 was purchased from Selleck Chemicals (Houston, TX, USA). HHT was purchased from Sigma-Aldrich (St. Louis, MO, USA). The antibodies GAPDH (#5174), PARP (#9532), Caspase3 and Cleaved-Caspase3 (Cleaved-C3) (#9662), Caspase7 and Cleaved-Caspase7 (Cleaved-C7) (#9494), PI3K (p85α) (#4257), AKT (#4691), AKT (Ser473) (#4060), GSK3β (#9315), GSK3β (Ser9) (#9323), MCL-1 (#94296), MCL-1 (Thr163) (#14765) and MCL-1 (Thr163/Ser159) (#4579) were purchased from Cell Signaling Technology (CST, Beverly, MA, USA).

### Cell lines and primary cells

MV4-11 cell line was kindly endowed by Professor R. Bhatia (City of Hope National Medical Center, Duarte, CA, USA). THP-1, HL-60, U937 and OCI-AML3 cell lines were purchased from the Shanghai Cell Bank of the Chinese Academy of Sciences. Kasumi-1 cell line was gifted by Professor Chen Saijuan (Shanghai Institute of Hematology, Shanghai, China). MV4-11 cells were maintained in Iscove’s Modified Dulbecco’s medium (IMDM, Gibco, Billings, MT, USA) supplemented with 10% fetal bovine serum (FBS, Gibco), and THP-1, HL-60, U937 and OCI-AML3 cells were cultured in Roswell Park Memorial Institute 1640 (RPMI 1640, Gibco) medium supplemented with 10% FBS at 37 °C in a humidified incubator containing 5% CO_2_.

Bone marrow samples were obtained from AML patients following written informed consent. Mononuclear cells were isolated by Ficoll-Hypaque (Sigma-Aldrich, St. Louis, MO, USA) density gradient centrifugation. The study protocol was approved by the Ethics Committee of the First Affiliated Hospital of Zhejiang University, China.

### Cell viability assay

20,000 AML cells (cell density of 2.0 × 10^5^ cells/ml) or 100,000 primary AML cells (cell density of 1.0 × 10^6^ cells/ml) were seeded in a 96-well plate and treated with designated drugs for the 24 or 48 h. Then 10 ul MTS solution (Promega CellTitre 96, Promega Corporation, Madison, WI, USA (5 mg/ml) was added by incubation for additional 3–4 h at 37℃. The plates were measured at an absorbance of 490 nm. IC50 values and combination index (CI) were calculated by CalcuSyn Software (Biosoft, Cambridge, UK) (CI: < 1, synergism; > 1, antagonism; = 1, additivity). That program was based on the following equation: q = E_A+B_ / (E_A_ + E_B_—E_A_ × E_B_), where E_A_ and E_B_ are the inhibition rate of group A and group B, respectively, and E_A+B_ is the inhibition rate of group A combined with B (q: > 1.15, synergism; < 0.85, antagonism; 0.85–1.15, additivity) [23, 24].

### Flow-cytometric analysis of apoptosis

The extent of apoptosis was assessed through an apoptosis detection kit (Beyotime Institute of Biotechnology, Hangzhou, China). Cells were treated with drugs for 24 h, and then harvested and washed with phosphate buffered saline (PBS). According to the manufacturer’s instruction, after resuspending in binding buffer and staining with Annexin V-FITC (Annexin-V) and propidium iodide (PI) in dark at room temperature for 15 min, cells were subjected to FACScan™ flow cytometer (Becton Dickinson, San Diego, CA, USA). Data were presented as percentages of Annexin V + cells.

### Western blot analysis

Cells were harvested and washed in PBS, and then lysed in radioimmunoprecipitation (RIPA) buffer in the presence of protease and phosphatase inhibitor (Cell Signaling Technology (CST), Beverly, MA, USA)) on ice for 30 min. Cells were centrifuged at 12,000*g* for 15 min at 4 °C to pellet cell debris. The protein concentration was determined using BCA reagent. Equal protein was electrophoresed in 10% SDS-PAGE (Life Technologies, Carlsbad, CA, USA) and transferred to a PVDF membrane (Millipore, Billerica, MA, USA). The transblotted membranes were blocked with 5% non-fat milk in TBS-T for 1 h and then incubated with primary antibodies overnight at 4 ℃. Then the membranes were washed thrice with TBS-T buffer, 15 min each time, followed by secondary HRP-conjugated antibody (CST, Beverly, MA, USA) for 1 h at room temperature. The target proteins were visualized using an ECL detection kit (Amersham, Little Chalfont, UK) and analyzed using IMAGE LAB^TM^ software (Bio-Rad Laboratories, Hercules, CA, USA).

### AML xenograft model

BALB/c Nude mice (female, 4–6 weeks of age, weight 17–24 g) were purchased from Shanghai Lingchang Biotechnology Co., Ltd. (Shanghai, China) and Hangzhou Medical College Laboratory animal center. MV4-11 cells were resuspended in PBS (1 × 10^6^ cells/mouse), and then subcutaneously injected into the right back of BALB/c Nude mice to establish a subcutaneous xenograft tumor model. When the tumor volume reached to 50–150 mm^3^, the mice were randomly divided into 6 groups (vehicle control, 50 mg/kg APG-2575, 50 mg/kg ABT-199, 1 mg/kg HHT, 50 mg/kg APG-2575 + 1 mg/kg HHT, 50 mg/kg ABT-199 + 1 mg/kg HHT). The administration was started on the day of grouping (ie D1; APG-2575/1BT-199, PO, QDx21D; HHT, IP, QDx14D). The mouse body weight and tumor size were measured twice a week during the experiment. After the administration, all mice were killed by cervical dislocation. In addition, OCI-AML3 cells were injected subcutaneously on the left back of BALB/c Nude mice, and the other operations referred to the above.

Tumor volume (TV) was calculated as: TV = L × W^2^/2, Where L and W represent the length and width of the tumor, respectively. The relative tumor volume (RTV) was: RTV = Vt/V1. V1 is the TV on the first day of administration (Day1) and Vt is the TV at the time of measurement (Day t). The evaluation index of anti-leukemic effect was the relative tumor proliferation rate: (T/C (%) = (T_RTV_/C_RTV_) × 100%) [T_RTV_: the RTV of the treatment group, C_RTV_: the RTV of the vehicle control group]. Synergy analysis was measured by using the formula [25]: expected value = (A/C) × (B/C) [actual value = (AB)/C; A: RTV of A drug; B: RTV of B drug; C = The RTV of the vehicle control group; AB = the RTV of the combined administration group]. The synergy index = expected value/actual value; ratio > 1, indicating that the two drugs have a synergistic effect; ratio = 1, indicating that the two drugs have an additive effect; ratio < 1, indicating that the two drugs have a weaker than additive effect. Change of body weight (%) = (measured weight-weight at grouping)/weight at grouping × 100%. In addition, complete regression (CR), partial regression (PR) and stable disease (SD) among the number of animals in each group represented the remission rate. T/C (%) ≤ 42% and statistical analysis P < 0.05 is effective. If the weight of the mouse drops by more than 20% or the number of drug-related death exceeds 20%, the drug dose is considered to be severely toxic.

All animal experiments were reviewed and approved by the Institutional Animal Care and Use Committee (IACUC) of Suzhou GenePharma (Approval No: IACUC-2021003) and Hangzhou Medical College Laboratory animal center (Approval No: ZJCLA-IACUC-20120003).

### Statistical analyses

The one-way ANOVA test were used to assess statistical significance. P value < 0.05 was considered statistically significant. Experiments were presented as the means ± standard deviation (SD) in three independent experiments. The results of animal experiments and cell inhibition assay in primary AML samples were presented as the means ± standard error of the mean (SEM). Statistical analyses were conducted using Prism software v8.3.0 (GraphPad Software, La Jolla, CA, USA) and IBM SPSS Statistics 24 (Armonk, NY, USA).

## Results

### APG-2575 shows comparable anti-leukemic effect to ABT-199

**Fig. 1:**
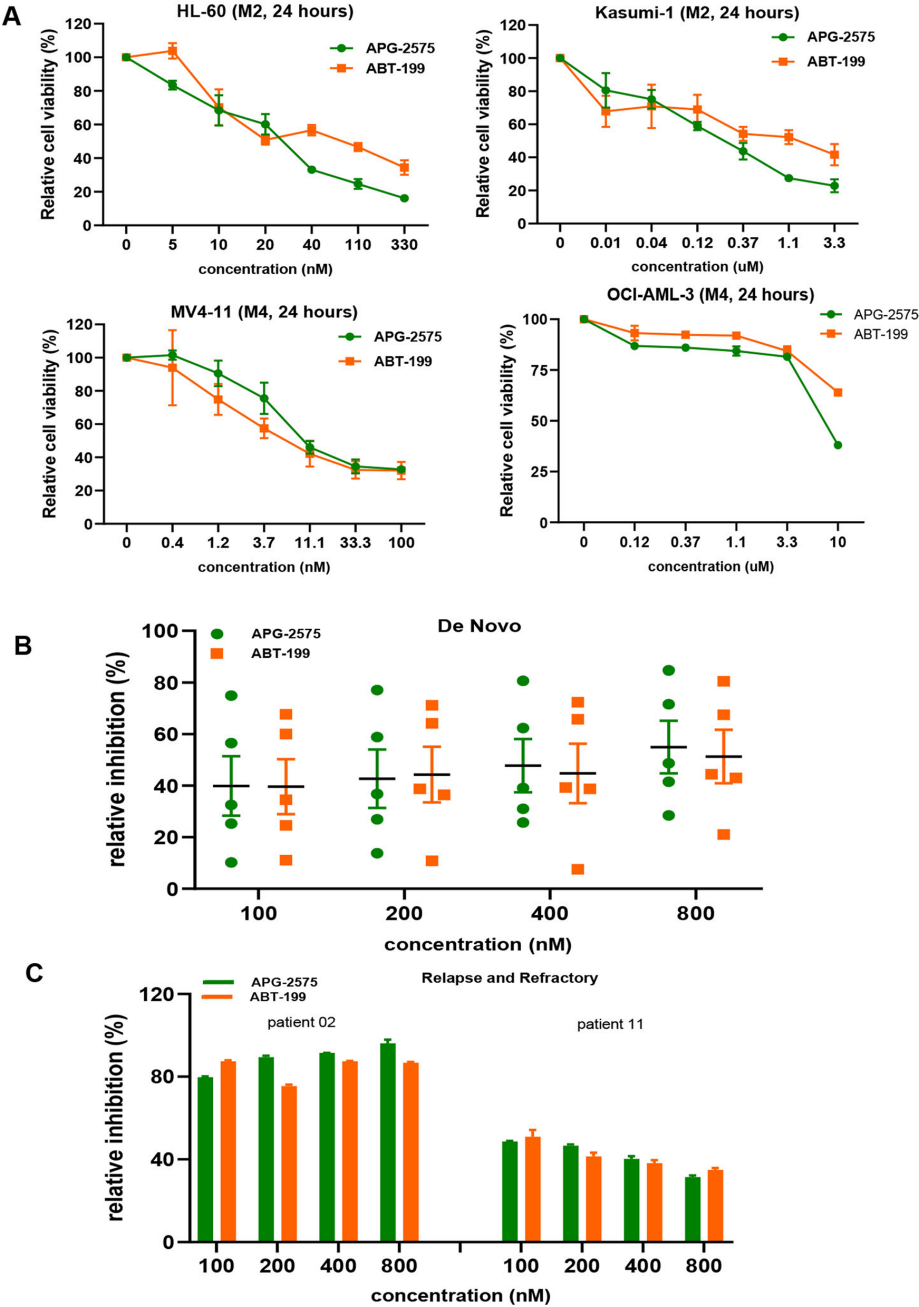
APG-2575 showed comparable anti-leukemic effect to ABT-199. **A** The cell viability of different concentrations of APG-2575 or ABT-199 in HL-60, Kasumi-1, MV4-11 and OCI-AML3 cells for 24 h. **B**, **C** The inhibition ratio of APG-2575 and ABT-199 in primary AML at different concentrations for 48 h

HL-60, Kasumi-1, MV4-11 and OCI-AML3 cells were treated with APG-2575 or ABT-199 for 24 h. The results showed that APG-2575 or ABT-199 treatment inhibited AML cells viability in a dose-dependent manner. The four AML cell lines showed similar response to APG-2575 and ABT-199 (Fig. 1A). The IC50 values of six cell lines (HL-60, Kasumi-1, MV4-11, THP-1, U937 and OCI-AML3) were determined after treatment with APG-2575 or ABT-199 for 24 h. The anti-leukemic activity of APG-2575 in AML cells was equal or even better than that of ABT-199 (Additional file 1: Fig. S1, Additional file 2: Table S1). Additionally, similar anti-leukemic effect of APG-2575 and ABT-199 was also found when they were used to treat primary AML samples from 5 newly diagnosed patient and 2 relapse/ refractory patients (Fig. 1B, C). The clinical characteristics of the patient samples were presented in Table 1.

**Table 1:** Clinical characteristics of primary AML patients

	Diagnose	Gender	Age (year)	FAB type	Cytogenetics	Molecular	WBC (*10^9/L)	HB (g/L)	PLT (*10^9/L)
patient 01	De novo	Female	69	M5b	46, XX	MLL-ELL, NRAS, KARS CSMD1, MECOM	68.87	87	29
patient 02	R/R	Female	74	M5	NA	FLT3-TID, NPM1	80.94	64	193
patient 03	De novo	Male	63	M2a	Complex karyotype	TP53, NARS	59.9	54	23
patient 04	De novo	Female	55	M5	46, XX	FLT3-TID, DNMT3A	165.11	78	115
patient 05	De novo	Female	44	M5	46, XX, del (8) (q22)	NA	12.79	66	10
patient 06	De novo	Male	34	M4	46, XY	FLT3-TID, DNMT3AIDH1	121.7	74	169
patient 07	De novo	Male	15	M5	Complex karyotype	FLT3, CBFβ-MYH11, NARS	31.6	79	22
patient 08	De novo	Female	50	M5	46, XX	FLT3-TID	24.57	70	105
patient 09	De novo	Female	43	M1	46, XX	WT1, FLT3, IDH2, NPM1	33.5	80	16
patient 10	De novo	Male	21	M4	47, XY, + mar	NA	28.17	45	4
patient 11	R/R	Female	44	M2a	NA	WT1, GATA2, CEBPA, KIT	11.45	52	15
patient 12	Refractory	Female	43	M4	46, XX, t (3; 3) (q21; q26)	NA	91.3	43	31

### HHT and APG-2575 synergistically inhibit AML cells growth

**Fig. 2:**
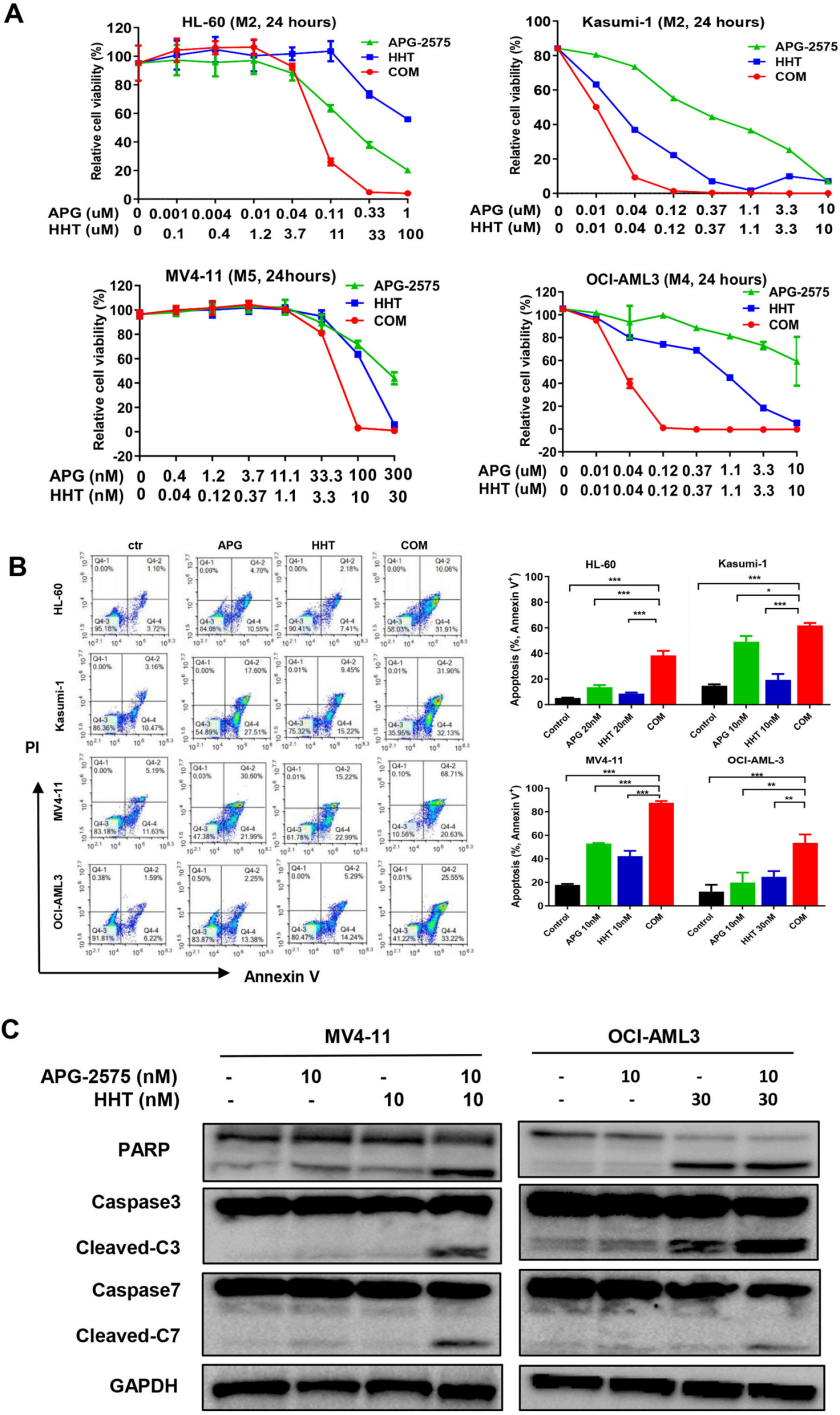
HHT and APG-2575 synergistically inhibit AML cells growth. **A** Cell viability of AML cell lines (HL-60, Kasumi-1, MV4-11 and OCI-AML3) treated with APG-2575 and HHT single agent or their combination for 24 h. **B** The apoptosis of HL-60, Kasumi-1, MV4-11 and OCI-AML3 cells treated with APG-2575 and HHT single agent or their combination for 24 h. (One-way ANOVA, combination treatments versus control and single treatments, *P < 0.05; **P < 0.01; ***P < 0.001). **C** The levels of apoptosis related proteins were determined after drug exposure for 24 h

To test the synergistic anti-leukemic activity between HHT and APG-2575, we treated several AML cells lines with HHT, APG-2575 and their combination at various concentrations and monitored cell viability. Remarkably, combine treatment of HHT with APG-2575 augmented the inhibitory effect to a greater extent than either of the two agents alone in HL-60, Kasumi-1, MV4-11 and OCI-AML3 cell lines (Fig. 2A, Additional file 2: Table S2).

Considering that APG-2575 is a BCL-2 inhibitor and exerts an anti-leukemic effect through the apoptotic pathway, we tested whether the combination treatment could synergistically trigger apoptosis in AML cells. HL-60, Kasumi-1, MV4-11 and OCI-AML3 cell lines were exposed to APG-2575 and HHT alone or in combination for 24 h. The Annexin-V/PI staining results indicated that APG-2575 induced AML cells apoptosis, and combination treatment resulted in superior induction of apoptosis compared with either single agent (Fig. 2B). Moreover, we analyzed the level of proteins involving apoptosis signaling pathway by western blot. Cleaved PARP, cleaved caspase-3 and cleaved caspase-7 were more significantly increased in the combined treatment group compared with the single-agent group (Fig. 2C), indicating HHT potentiates APG-2575 induced apoptosis in AML cells.

### HHT and APG-2575 combination exerts synergistically anti-leukemic effect in primary AML samples

**Fig. 3:**
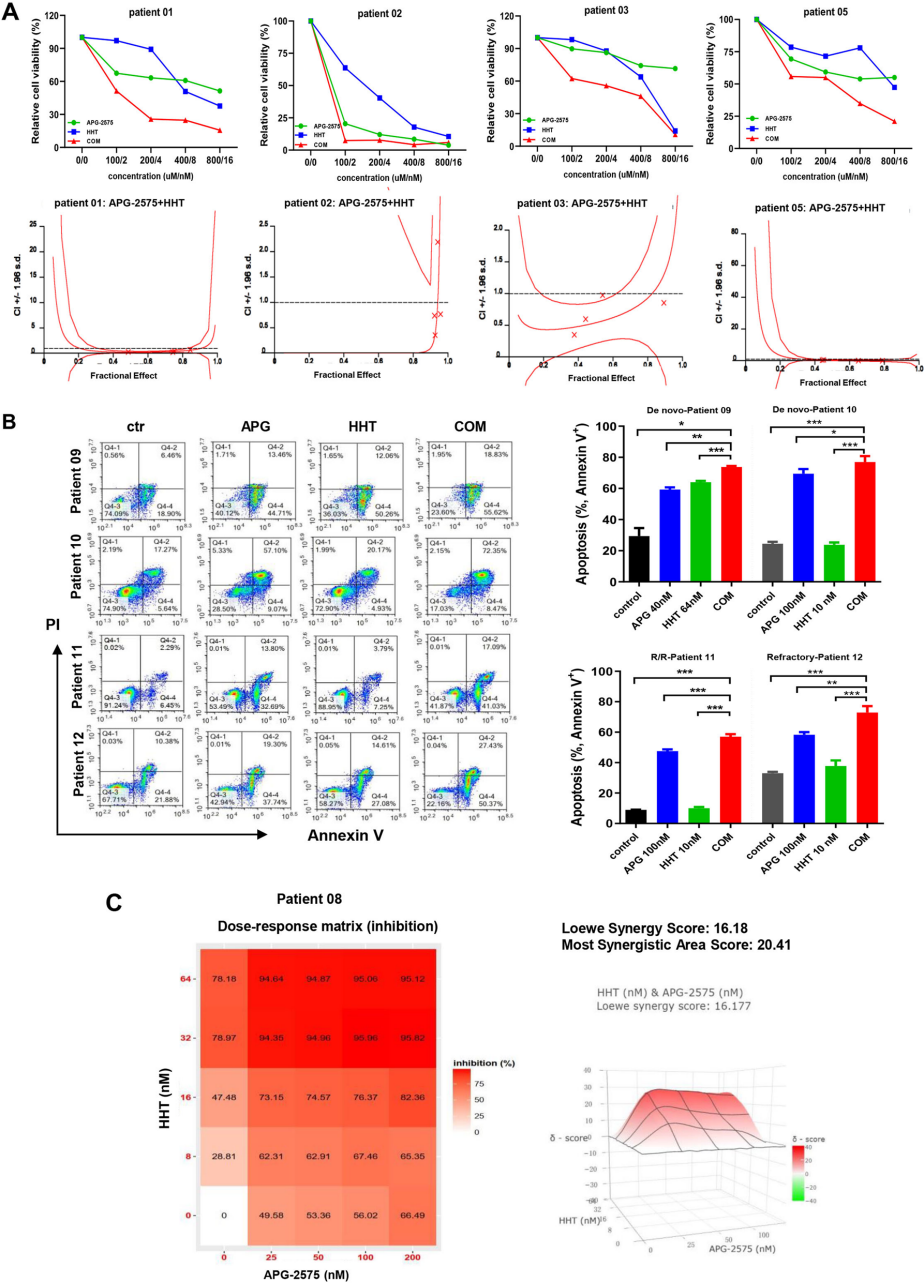
HHT and APG-2575 combination exerts synergistically anti-leukemic effect in primary AML samples. **A** Cell viability of AML patients treated with APG-2575 or HHT single agent or their combination for 48 h. The CI indexes (down panel) were calculated by CalcuSyn software. **B** The apoptosis of primary AML samples treated with APG-2575 and HHT single agent or their combination for 24 h. (One-way ANOVA, combination treatments versus control and single treatments, *P < 0.05; **P < 0.01; ***P < 0.001). **C** Cell viability was calculated for every dose combination of APG-2575 and HHT using the SynergyFinder Web application. Synergy score: < − 10, antagonistic; from − 10 to 10, additive; > 10, synergistic

We further validated the synergistic effect of HHT and APG-2575 combination in primary AML patient samples. The results revealed that APG-2575 or HHT can inhibit the viability of primary AML cells, and indeed, synergies (CI < 1.0) were also observed in AML specimens (Fig. 3A, Additional file 2: Table S3). The Annexin-V/PI staining results suggested that APG-2575 combined with HHT could induce significant primary AML cells (De novo or R/R) apoptosis compared with the single agent (Fig. 3B). Furthermore, inhibition rate was presented for every dose combination of HHT and APG-2575 using the Synergy Finder application (https://synergyfinder.fimm.fi). Synergy score was calculated based on the Loewe model. Dose-dependent inhibition of proliferation was observed upon HHT or APG-2575 treatment for 48 h, according to the overall drug combination dose–response matrix pattern (Fig. 3C). The average synergy score was 16.18 and the most synergistic area score was 20.41 in AML patient 08. These results indicated HHT combined with APG-2575 exerted a synergistically anti-leukemic effect in primary AML samples. The characteristics of the AML patients were presented in Table 1.

### APG-2575 synergized with HHT suppresses AML xenograft tumor growth

**Fig. 4:**
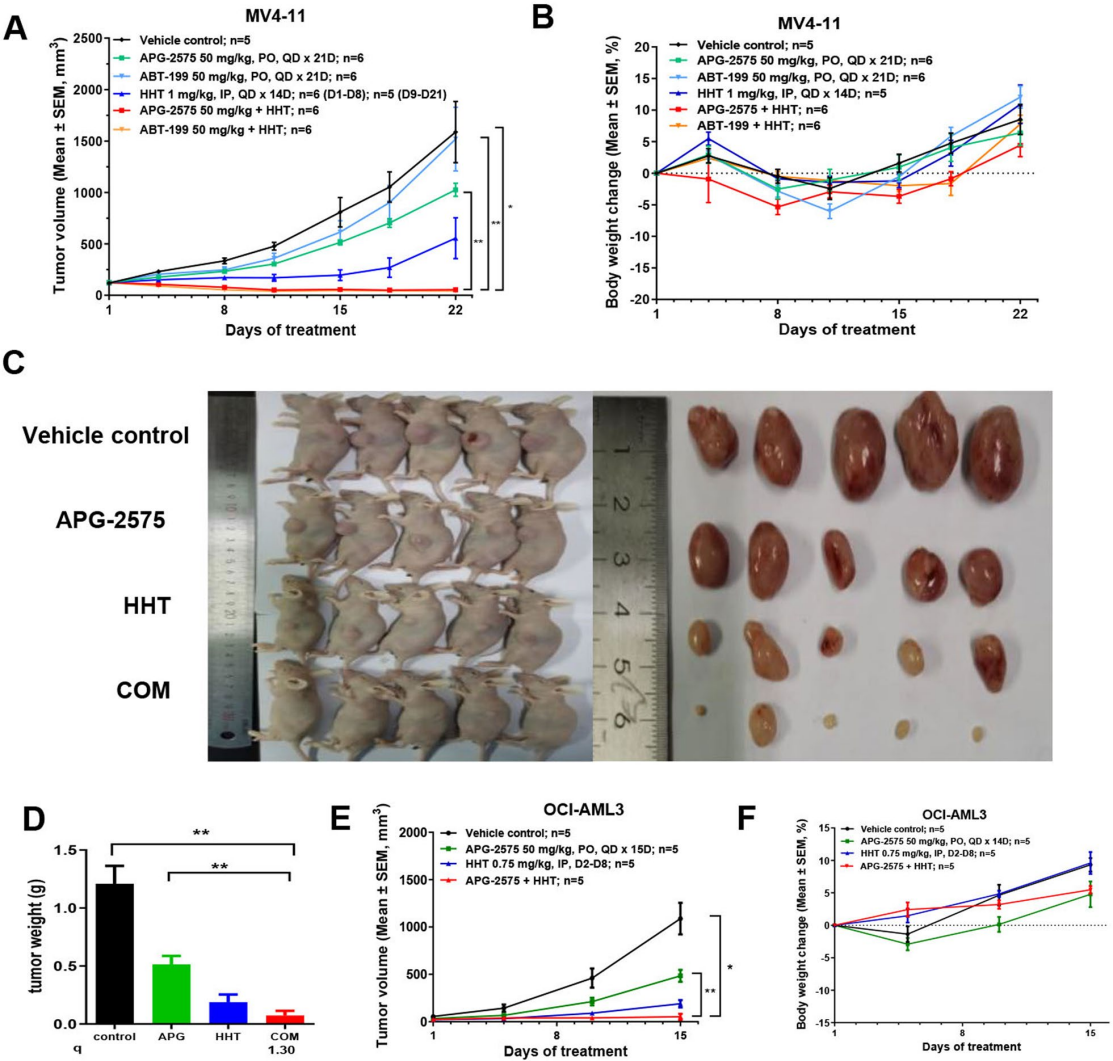
APG-2575 synergized with HHT suppresses AML xenograft tumor growth. **A**, **B** The average tumor volume cure and the body weight change curve in MV4-11 xenograft mouse model for each group. **C** Images of OCI-AML3 xenograft tumors (n = 5). **D–F** The tumor weight, average tumor volume curve and the body weight change curve in OCI-AML3 xenograft mouse model. (One-way ANOVA, combination treatments *versus* control and single treatments, *P < 0.05; **P < 0.01; ***P < 0.001)

To evaluate the anti-leukemic effect of the combination of BCL-2 inhibitor and HHT in vivo, we established AML xenograft mouse model (Fig. 4). The APG-2575 and HHT monotherapy group showed some anti-leukemic effect in MV4-11 xenograft mouse model, with T/C values of 62.76% and 32.43%, respectively, however, the APG-2575/HHT co-treatment group presented a significant synergistic anti-leukemic effect, with a T/C value of 3.31%. The synergy index was 6.14 and tumor remission rate was 100% (6/6 PR). In parallel comparison, another FDA-approved BCL-2 inhibitor ABT-199 combined with HHT also showed an obvious synergistic anti-AML effect, with a T/C value of 2.59%. The synergy index was 11.07 and the tumor remission rate was 100% (6/6 PR) (Fig. 4A, Additional file 2: Table S4). These results suggested that APG-2575 combined with HHT had a significant synergistic anti-AML effect, and the synergistic effect was comparable to that of ABT-199 combined with HHT. No significant body weight loss was observed in the animals in each administration group (Fig. 4B), indicating that the animals were well tolerated the compounds.

OCI-AML3 cells were used to establish a mouse subcutaneous xenograft tumor model as well. OCI-AML3 cell was characterized by a rapidly growing xenograft tumor. On the 15th day after administration, the animals in the vehicle control group were obviously anxious and restless. Therefore, the experiment was terminated on the 15th day. APG-2575 and HHT single agent did not show significant antitumor activity in this model, with a T/C value of 75.44% and 41.95%, respectively (Fig. 4C, E, Additional file 2: Table S5). Notably, the co-treatment group had significant synergistic anti-leukemic effect with T/C value of 8.52% (compared with the vehicle control group, P < 0.001). The synergy index was 3.71 (Additional file 2: Table S5). Furthermore, the tumor weights of the APG-2575 and HHT single agent treated groups were lower than the vehicle control group, while the co-treatment group showed the lowest tumor burden among four groups (Fig. 4D). The q value (q = 1.30) indicated HHT combined with APG-2575 exerted a synergistically anti-leukemic effect. No significant body weight loss was observed in the animals in each group (Fig. 4F).

### HHT reverses the resistance of APG-2575 via inhibiting the PI3K/AKT/GSK3β pathway

**Fig. 5:**
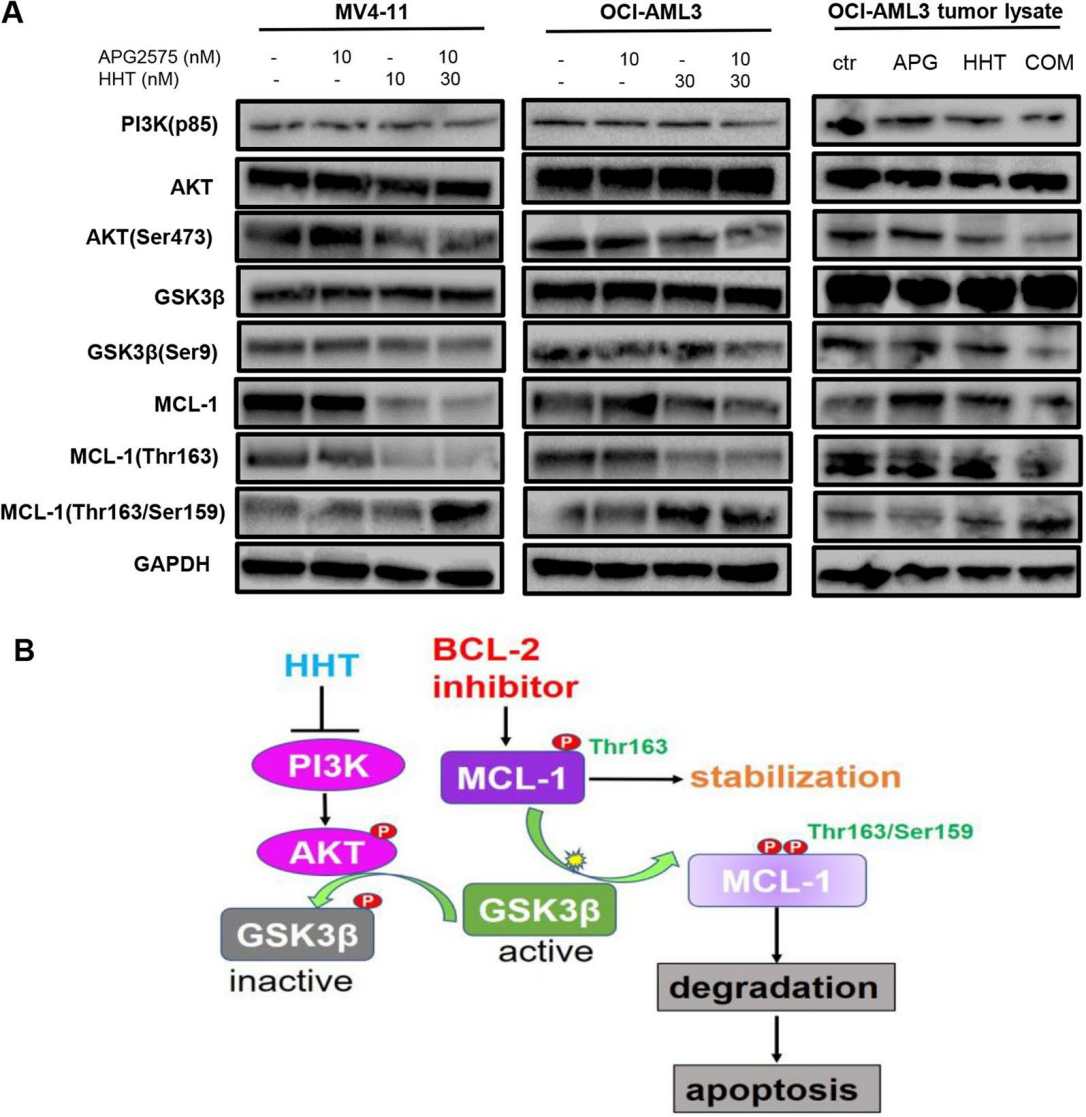
HHT reverses the resistance of APG-2575 via inhibiting the PI3K/AKT/GSK3β pathway. **A** The level of proteins in PI3K/AKT/GSK3β signaling pathway and the MCL-1(Thr163, Thr163/Ser159) were measured after treatment with APG-2575 and HHT for 4 h. And the MCL-1 for 6 h. (in vitro and in vivo). **B** Schematic of APG-2575 and HHT in AML. HHT inhibits the PI3K/AKT/GSK3β pathway, which interfere with MCL-1 stability, thereby accelerating MCL-1 degradation

As MCL‐1 is a vital player in the resistance to BCL-2 inhibitor in AML cells. We examined the expression of MCL-1 protein after exposure to APG-2575 or HHT alone or combined regimen. Western blot analysis revealed that, while exposure to APG-2575 lead to increase MCL-1 expression, treatment with HHT alone or the combination results in a significant reduction in the protein level of MCL-1 in MV4-11, OCI-AML3 cells. It was reported that GSK3β was involved in MCL-1 degradation [26–29]. We next examined whether the combination of APG-2575 and HHT affects the PI3K/AKT/GSK3β signaling pathway. As shown in Fig. 5 A, HHT in the presence or absence of APG-2575 led to a reduction of PI3K, p-AKT (Ser473), and p-GSK3β (Ser9) expression, but has no obvious effect on the total protein level of AKT and GSK3β. Of note, our study indicated that APG-2575 showed no effects on PI3K/AKT/GSK3β signaling pathway. In addition, monophosphorylated and dual phosphorylated MCL-1 were detected by western blot. The results suggested that HHT could reduce p-MCL-1 (Thr163) and up-regulate p-MCL-1 (Thr163/Ser159), and notably, this effect of HHT was enhanced in the combination treated cells. We also verified that APG-2575 combined with HHT inhibited the PI3K/AKT/GSK3β signaling pathway and promoted MCL-1 degradation in vivo (Fig. 5A). Treatment with APG-2575 increased MCL-1 level and HHT promoted the phosphorylation and degradation of MCL-1 via inhibiting the PI3K/AKT/GSK3β signaling pathway, thereby reversing the resistance of APG-2575 (Fig. 5B).

## Discussion

AML is a genetically heterogeneous disorder characterized by a poor survival rate. Regardless of whether regimens combine conventional therapies with multiple novel target drugs or hematopoietic stem cell transplantation (HSCT), AML remains an incurable malignancy of the bone marrow. New regimens with better tolerance and efficacy, less toxicity and fewer side effects are urgently needed. On June 7, 2021, Ascentage Pharma Ascent Pharmaceuticals presented the latest clinical progress of the new generation of Bcl-2 inhibitor APG-2575 at the ASCO meeting. Among 15 patients with R/R CLL/SLL, 12 cases achieved partial remission (PR) and the overall remission rate (ORR) was as high as 80.0%. No dose-limiting toxicity (DLT) was observed at 1200 mg and no tumor lysis syndrome occurred [15]. It means that there are great potentials for clinical development of single drug and combination therapy of APG-2575, which will bring better options for patients. MCL-1 plays a vital role in the development of hematological malignancies and high expression of MCL-1 is associated with BCL-2 inhibitor resistance. Our previous studies identified that HHT could reduce MCL-1 and exert significant anti-leukemic activity [26, 27]. Therefore, we propose the combined application of APG-2575 and HHT for the treatment of AML in order to reverse the resistance of APG-2575.

In this study, we first compared the anti-leukemic effect of APG-2575 and ABT-199 and the results suggested that the two agents were comparable in inhibiting AML cells growth. Next, we found HHT in combination with APG-2575 displayed an excellent anti-leukemic effect. APG-2575 showed dose-dependent growth inhibition and accelerate apoptosis when administrated as a monotherapy, and the combined regimen exhibited more obvious synergistic activity, as evidenced by the activation of PARP and caspase family members. Studies had shown that tumor cells with high expression of anti-apoptotic protein MCL-1 have primary resistance to the BCL-2 selective inhibitor [28]. Interestingly, the two agents exerted more significant growth inhibition and apoptosis in OCI-AML3 cells (inherent resistance to APG-2575) and AML patient samples (De novo or R/R). These results demonstrated that HHT enhanced the anti-leukemic effect of APG-2575, suggesting HHT as a potential drug to overcome the resistance of APG-2575.

According to the cell experiments in vitro, MV4-11 cells are moderately sensitive to APG-2575. Therefore, we first used human MV4-11 cells to establish a mouse xenograft tumor model to evaluate the anti-tumor effect of the combination of APG-2575 and HHT. The results suggested that APG-2575 and ABT-199 have equally anti-leukemic effects in vivo. The data proved that APG-2575 combined with HHT had a synergistic effect in vivo*.* And we further confirmed co-administration of APG-2575/HHT also exerting synergistic anti-leukemic effect in the OCI-AML3 xenograft model. Together, these above results verified that the combination of APG-2575 and HHT has a significant synergistic anti-AML effect in vitro and in vivo, indicating that this combined regimen could bring clinical benefits to AML patients.

GSK3β has been reported to be related to p-MCL-1 (Thr163/Ser159), and the double-phosphorylated MCL-1 (Thr163/Ser159) is degraded through the ubiquitinase pathway. Phosphorylation at serine 9 weakens GSK3β constitutive activity [29, 30]. Our results suggested that single-agent APG-2575 could not down-regulate p-GSK3β (S9), thereby preventing the conversion of p-MCL-1 (Thr163) to p-MCL-1(Thr163/Ser159). The accumulated p-MCL-1 (Thr163) could stabilize the structure of MCL-1 and made MCL-1 being more difficult to be degraded. APG-2575 combined with HHT can inhibit the PI3K/AKT/GSK3β signaling pathway. Because p-GSK3β (Ser9) is down-regulated, it was beneficial to the conversion of mono-phosphorylated p-MCL-1(Thr163) to double-phosphorylated p-MCL-1(Thr163/Ser159), and further degradation through ubiquitinase. The inhibition of the signaling pathway leads to the decrease of MCL-1 content and reverses the resistance of APG-2575.

## Conclusion

Some studies have reported that high expression of MCL-1 was related to non-remission following ABT-199 treatment, and the original resistance in AML cells could reverse by co-administration of MCL-1 inhibitor. In this study, HHT combined with APG-2575 treatment could reduce the level of MCL-1. Overall, HHT combined with APG-2575 could overcome the resistance to APG-2575 in AML, and these preclinical results provided strong evidence for the treatment of AML patients with APG-2575 combined with HHT.

## Supplementary Information

**Additional file 1: Figure S1.** The IC50 values of APG-2575 or ABT-199 in HL-60, Kasumi-1, MV4-11, THP-1, U937 and OCI-AML3 for 24 h.

**Additional file 2: Table S1.** IC50 values of APG-2575 and ABT-199 against AML cell lines. **Table S2.** The CI and q values of AML cell lines. **Table S3.** The combination index values of AML primary patients. **Table S4.** The evaluation indicators of tumor bearing mice in MV4-11 model. **Table S5.** The evaluation indicators of tumor bearing mice in OCI-AML3 model.

## Data Availability

The datasets generated during the current study are not publicly available due confidentiality for another study but are available from the corresponding author on reasonable request.
