# Supplementary figures and images for: Homoharringtonine is synergistically lethal with BCL-2 inhibitor APG-2575 in acute myeloid leukemia
Source: J Transl Med. 2022 Jul 6;20:299. doi: 10.1186/s12967-022-03497-2 (PMC9258085; doi:10.1186/s12967-022-03497-2)

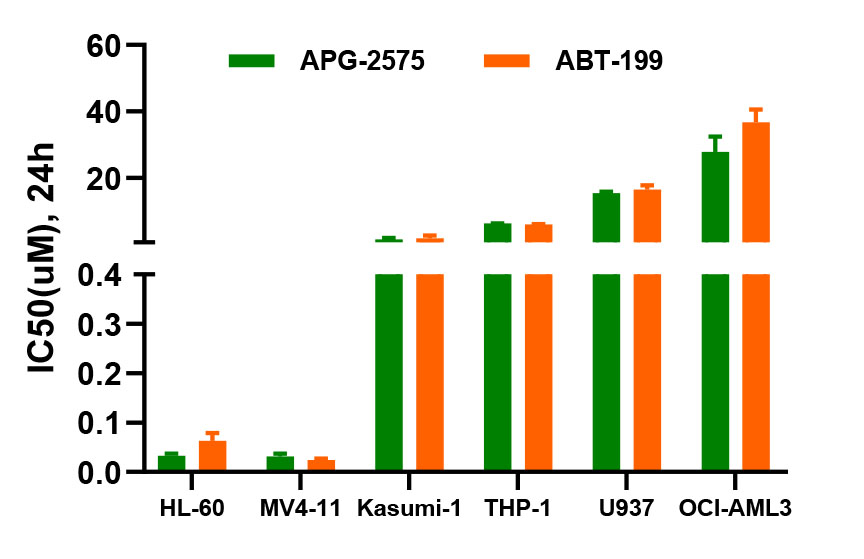

Supplement: Supplementary file 1 — Additional file 1: Figure S1. The IC50 values of APG-2575 or ABT-199 in HL-60, Kasumi-1, MV4-11, THP-1, U937 and OCI-AML3 for 24 h. [file 12967_2022_3497_MOESM1_ESM.jpg]
