# Supplementary material for: Homoharringtonine is synergistically lethal with BCL-2 inhibitor APG-2575 in acute myeloid leukemia
Source: J Transl Med. 2022 Jul 6;20:299. doi: 10.1186/s12967-022-03497-2 (PMC9258085; doi:10.1186/s12967-022-03497-2)
Supplement: Supplementary file 2 — Additional file 2: Table S1. IC50 values of APG-2575 and ABT-199 against AML cell lines. Table S2. The CI and q values of AML cell lines. Table S3. The combination index values of AML primary patients. Table S4. The evaluation indicators of tumor bearing mice in MV4-11 model. Table S5. The evaluation indicators of tumor bearing mice in OCI-AML3 model. [file 12967_2022_3497_MOESM2_ESM.docx]

Supplemental materials for “**Homoharringtonine is** **Synergistically Lethal with BCL-2 inhibitor APG-2575 in Acute Myeloid Leukemia**”

Additional Tables

**Table S1.** IC50 values of APG-2575 and ABT-199 against AML cell lines

| FAB type | Cell Line | APG-2575 IC_50_ | | ABT-199 IC_50_ | |
| --- | --- | --- | --- | --- | --- |
|  |  | (μM) [Mean (SD)] | | (μM) [Mean (SD)] | |
| M2 | HL-60 | 0.03 | 0.01 | 0.06 | 0.02 |
| M5 | MV4-11 | 0.03 | 0.01 | 0.02 | 0.00 |
| M2 | Kasumi-1 | 1.45 | 0.55 | 1.75 | 1.12 |
| M5 | THP-1 | 6.19 | 0.07 | 5.92 | 0.13 |
| M4 | U937 | 15.35 | 0.53 | 16.46 | 1.79 |
| M4 | OCI-AML3 | 27.77 | 6.55 | 36.73 | 5.39 |

**Table S2.** The CI and q values of AML cell lines.

| **HL-60** |  |  |  |  |  | |  | |  | |  |
| --- | --- | --- | --- | --- | --- | --- | --- | --- | --- | --- | --- |
| HHT (uM) | 0.1 | 0.4 | 1.2 | 3.7 | 11 | | 33 | | 100 | |  |
| APG (uM) | 0.001 | 0.004 | 0.01 | 0.04 | 0.11 | | 0.33 | | 1.00 | |  |
| CI | - | - | - | - | 0.114 | | 0.028 | | 0.067 | |  |
| q | - | - | - | - | 1.980 | | 1.307 | | 1.081 | |  |
| **Kasumi-1** |  |  |  |  |  | |  | |  | |  |
| HHT (uM) | 0.01 | 0.04 | 0.12 | 0.37 | 1.1 | | 3.3 | | 10 | |  |
| APG (uM) | 0.01 | 0.04 | 0.12 | 0.37 | 1.1 | | 3.3 | | 10 | |  |
| CI | - | 0.047 | 0.002 | 0.001 | 0.001 | | 0.000 | | 0.001 | |  |
| q | - | 1.244 | 1.124 | 1.027 | 1.004 | | 1.025 | | 1.004 | |  |
| **MV4-11** |  |  |  |  | |  | |  | |  | |
| HHT (nM) | 0.04 | 0.12 | 0.37 | 1.1 | | 3.3 | | 10 | | 30 | |
| APG (nM) | 0.4 | 1.2 | 3.7 | 11.1 | | 33.3 | | 100 | | 300 | |
| CI | - | - | - | - | | - | | 0.012 | | 0.009 | |
| q | - | - | - | - | | - | | 1.757 | | 1.013 | |
| **OCI-AML3** |  |  |  |  | |  | |  | |  | |
| HHT (uM) | 0.01 | 0.04 | 0.12 | 0.37 | | 1.1 | | 3.3 | | 10 | |
| APG (uM) | 0.01 | 0.04 | 0.12 | 0.37 | | 1.1 | | 3.3 | | 10 | |
| CI | 0.672 | 0.051 | 0.010 | 0.000 | | 0.000 | | 0.000 | | 0.010 | |
| q | 5.389 | 2.400 | 3.774 | 2.580 | | 1.580 | | 1.159 | | 1.035 | |

**Table S3.** The combination index values of AML primary patients

| CI | ED50 | ED75 | ED90 |
| --- | --- | --- | --- |
| patient 01 | 0.22006 | 0.38335 | 0.92697 |
| patient 02 | 1.79E-05 | 0.00131 | 0.09882 |
| patient 03 | 0.54117 | 0.82208 | 1.34239 |
| patient 05 | 0.30568 | 0.0932 | 0.04407 |

ED50: 50% effective dose; ED75: 75% effective dose; ED90: 90% effective dose

**Table S4.** The evaluation indicators of tumor bearing mice in MV4-11 model.

|  | **TV (D22)**  **（Mean ± SEM)** | **T/C (%)**  **（D22）** | **synergy index ^a^** | **mRECIST** |
| --- | --- | --- | --- | --- |
| Vehicle control | 13.33 ± 2.40 | - | - | 5/5 PD |
| APG-2575 50 mg/kg | 8.36 ± 0.76 | 62.76 | - | 6/6 PD |
| ABT-199 50 mg/kg | 11.77 ± 1.94 | 88.27 | - | 6/6 PD |
| HHT 1 mg/kg | 4.32 ± 1.42 | 32.43 | - | 3/5 SD,2/5 PD |
| APG-2575 + HHT | 0.44 ± 0.04^*##^ | 3.31 | 6.14 | 6/6 PR |
| ABT-199 + HHT | 0.34 ± 0.02^*$$^ | 2.59 | 11.07 | 6/6 PR |

^*^, compared to vehicle control, P < 0.05；^##^, compared to APG-2575，P < 0.01；^$$^, compared to ABT-199，P < 0.01；^a^ synergy index：>1 synergistic, = 1 additive, <1 antagonistic.

**Table S5.** The evaluation indicators of tumor bearing mice in OCI-AML3 model.

|  | **TV (D15) (Mean ± SEM**) | **T/C (%) (D15)** | **synergy index ^a^** |
| --- | --- | --- | --- |
| Vehicle control | 20.18 ± 1.25 | - | - |
| APG-2575 50 mg/kg | 15.22 ± 197 | 75.44 | - |
| HHT 0.75 mg/kg | 8.46 ± 1.536 | 41.95 | - |
| APG-2575 + HHT | 1.72 ± 0.63^***###^ | 8.52 | 3.71 |

^***^, compared to vehicle control, P < 0.001；^###^, compared to APG-2575，P < 0.001；^a^ synergy index：>1 synergistic, = 1 additive, <1 antagonistic.
